# A nomogram for malignancy prediction of pancreatic cystic lesions based on trans-abdominal ultrasound features

**DOI:** 10.1186/s12880-026-02325-z

**Published:** 2026-04-02

**Authors:** Liyuan Ma, Ya Hu, Yu Xia, Jiang Ji, Jionghui Gu, Nengwen Luo, Aonan Pan, Yang Cao, Yuang An, Luying Gao, Yuxin Jiang

**Affiliations:** 1 Department of Ultrasound, State Key Laboratory of Complex Severe and Rare Diseases, Peking Union Medical College Hospital, Chinese Academy of Medical Sciences and Peking Union Medical College, 1 Shuaifuyuan, Dongcheng District, Beijing, 100730 China; 2 Department of General Surgery, State Key Laboratory of Complex Severe and Rare Diseases, Peking Union Medical College Hospital, Chinese Academy of Medical Sciences and Peking Union Medical College, Beijing, 100730 China

**Keywords:** Pancreatic cystic lesions, Trans-abdominal ultrasound, Nomogram, Malignancy, Ultrasound

## Abstract

**Background:**

Pancreatic cystic lesions (PCLs) have variable malignant potential, and distinguishing benign from malignant/premalignant cysts is challenging for optimal management. Trans-abdominal ultrasound (TAUS), a widely available, non-invasive, low-cost first-line pancreatic imaging tool, is underexplored for predicting PCL malignant potential. This study aimed to develop a TAUS feature-based nomogram for this purpose.

**Methods:**

This retrospective study included 161 patients with pathology-confirmed PCLs from December 2012 to July 2021, divided into benign (59 cases) and non-benign (premalignant/malignant, 102 cases) groups. Relevant clinical characteristics and TAUS features were collected. Least absolute shrinkage and selection operator (LASSO) logistic regression analysis was used to optimize feature selection. Multivariate logistic regression analysis was applied to construct the nomogram. The performance of the nomogram was assessed via receiver operating characteristic curves, calibration curves and decision curve analysis (DCA).

**Results:**

Among the 26 features collected, 11 features were chosen via LASSO analysis. Multivariate analysis identified echogenicity, the configuration of cysts, solid content and septation/wall thickening as independent predictors. The prediction nomogram model developed with these four variables showed moderate discriminative performance in differentiating non-benign from benign PCLs, with an area under the curve (AUC) of 0.781. Regarding internal verification, tenfold cross-validation yielded a C-index of 0.749. The Hosmer–Lemeshow test yielded a *P* = 0.945, suggesting that the model had a good fit. Additionally, DCA demonstrated good net clinical benefit.

**Conclusions:**

This study explored the value of TAUS features for predicting the malignant potential of PCLs. The incorporation of TAUS features into a nomogram may offer a potential non-invasive tool to help clinicians in risk stratification during the initial evaluation and follow-up assessment of PCLs patients.

**Supplementary Information:**

The online version contains supplementary material available at 10.1186/s12880-026-02325-z.

## Background

Pancreatic cystic lesions (PCLs) refer to well-defined lesions in the pancreas that contains fluid, with increasing detection rates due to the widespread use of abdominal imaging techniques [1–3]. PCLs encompass a heterogeneous group of pathological entities, ranging from benign cysts to premalignant or malignant neoplasms [4, 5]. Key subtypes include serous cystic neoplasms (SCNs), mucinous cystic neoplasms (MCNs), intraductal papillary mucinous neoplasms (IPMNs), solid pseudopapillary neoplasms (SPNs), and pseudocysts [5, 6]. Risk stratification is critical, as IPMNs, MCNs and SPNs harbor malignant potential, while SCNs and pseudocysts typically follow a benign course [6–10]. The development of appropriate management strategies can be challenging due to the difficulty in distinguishing potentially malignant lesions from benign lesions, as well as in assessing the risk for malignant progression over time [2].

Currently, the preoperative diagnosis and risk stratification of PCLs rely on a combination of imaging modalities and invasive procedures, including computed tomography (CT), magnetic resonance imaging (MRI), endoscopic ultrasound (EUS) with fine-needle aspiration, and cyst fluid analysis [2, 11]. These modalities help in characterizing cyst morphology, communication with pancreatic ducts, and detecting worrisome features [11]. Based on these investigations, the guidelines recommended surgical intervention for PCLs with high-risk features (e.g., mural nodules, main duct dilation, growth rate ≥ 5 mm/2 years, cyst diameter ≥ 30 mm, or cytological atypia) and surveillance for undefined cystic lesions [3, 5]. However, these modalities present limitations: CT involves radiation exposure, MRI availability is restricted in resource-limited settings, and EUS is invasive, operator-dependent, and costly.

Transabdominal ultrasound (TAUS) offers distinct advantages, including real-time imaging, absence of radiation, low cost, and widespread accessibility, thus making it an attractive option for the initial evaluation and follow-up of patients with PCLs [12]. The characteristic features visualized on TAUS, such as cyst size, septations, configuration, connection to the main pancreatic duct and echogenicity, can provide valuable information. Modern high-resolution ultrasound with Doppler further enhances its capability to evaluate the structure and vascularity of pancreatic lesions [13]. While multiple studies have validated the value of CT/MRI for predicting the malignant potential of PCLs [14, 15], TAUS—a first-line imaging tool for pancreatic evaluation—remains underexplored for this purpose.

In this study, we aimed to develop a nomogram based on TAUS features to noninvasively predict the malignant potential of PCLs from a retrospective cohort.

## Methods

### Enrolment of study population

**Fig. 1:**
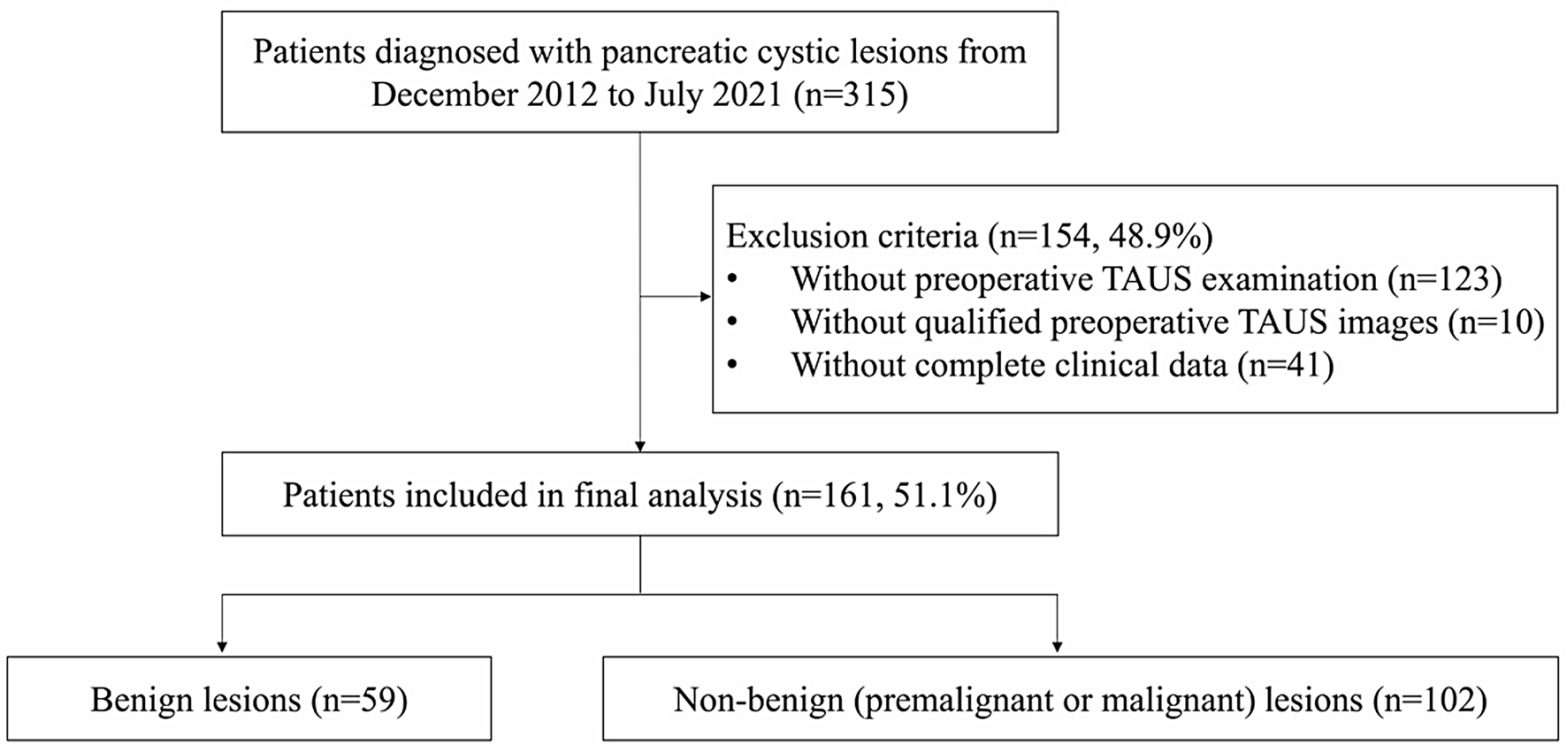
Flow chart of the process of patient enrollment. TAUS: Trans-abdominal ultrasound

This retrospective study was approved by the Ethics Committee of our hospital (No. K4829), and the requirement for written informed consent was waived. As Fig. 1 shows, a total of 315 patients who were diagnosed with PCLs at our hospital from December 2012 to July 2021 were collected. The diagnosis was made on the basis of pathology of the surgical specimens. Patients without preoperative TAUS examination, qualified preoperative TAUS images, and complete clinical data were excluded.

A total of 161 PCLs patients were finally included, and their clinical data were collected, including gender, age, body mass index (BMI), symptoms (such as abdominal pain, jaundice, nausea, vomiting, abdominal discomfort and distention, weight loss, and back pain), pancreatitis, diabetes mellitus, smoking, drinking, carcinoembryonic antigen (CEA), cancer antigen 199 (CA199), total bilirubin (TBil), and direct bilirubin (DBil) levels.

According to the European evidence-based guidelines [5], the clinical management of PCLs differs fundamentally based on their biological nature. Benign lesions such as SCN require only 1-year surveillance followed by symptom-based follow-up once definitively diagnosed, whereas all premalignant and malignant lesions require either surgical resection or lifelong imaging surveillance at 6–12 month intervals. Based on this distinction, we classified PCLs into benign and non-benign groups according to final pathological diagnosis. The benign group included SCNs, pseudocysts, lymphangiomas, and retention cysts, and the non-benign group included IPMNs, MCNs, SPNs, cystic neuroendocrine tumors (cNETs), and pancreatic ductal adenocarcinomas (PDACs) with cystic degeneration [8].

### Ultrasonic data collection

TAUS was performed using Philips IU22 or EPIQ7 ultrasound systems equipped with a 2–5 MHz convex array transducer. All examinations were conducted in the morning after an overnight fast of at least 8 h to minimize bowel gas interference. Patients were examined in the supine position, supplemented by lateral decubitus positions and breathing cooperation as needed for optimal visualization. The entire pancreas was systematically scanned from head to tail. B-mode imaging parameters including depth, overall gain, time-gain compensation, and focus were optimized to ensure adequate visualization of features of the lesion. Cyst dimensions were measured in three orthogonal planes: transverse and anteroposterior diameters on axial cross-sections, and longitudinal diameter on sagittal sections. The maximum diameter in any plane was recorded as the cyst size. Color Doppler imaging was performed with the velocity scale set to 10–15 cm/s, the sampling box adjusted to encompass the entire lesion with surrounding parenchyma, and the wall filter set to the lowest available level to optimize detection of low-velocity flow within solid components.

### Ultrasound image analysis

All TAUS images and reports were independently reviewed by two specialists (Reader 1 with over 20 years of experience and Reader 2 with over 10 years of experience) who were blinded to pathological results and each other’s assessments. To evaluate interobserver reliability, Cohen’s kappa (κ) was calculated for categorical variables and intraclass correlation coefficient (ICC, two-way random, absolute agreement) for continuous variables. Discrepancies were resolved by consensus, and the consensus results were used for subsequent analyses. Detailed interobserver agreement data are provided in Supplementary File 1.

**Fig. 2:**
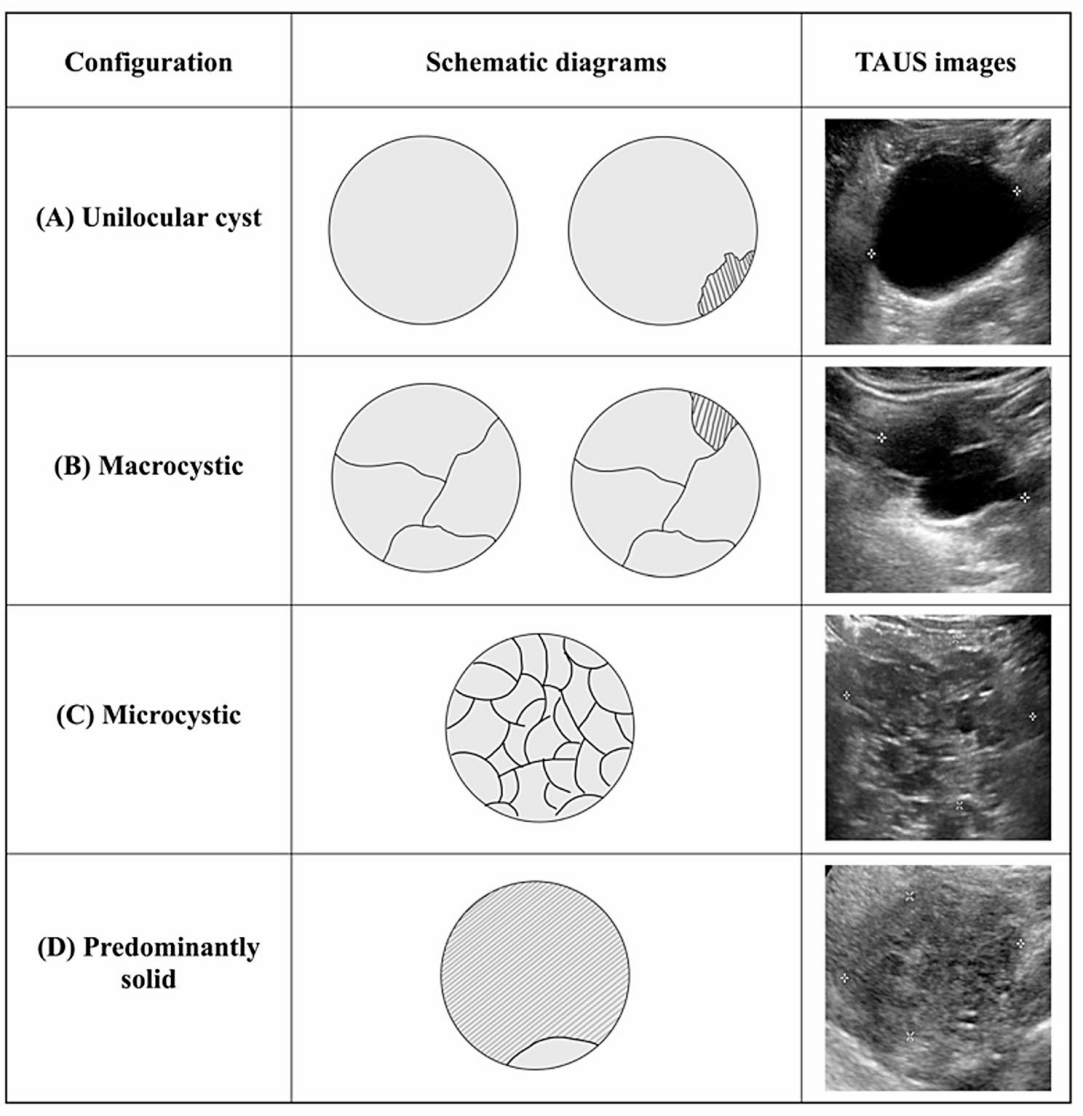
Schematic diagrams and representative TAUS images of the four cyst configuration categories. (**A**) Unilocular cyst: a single cystic compartment without internal septations. (**B**) Macrocystic: multiple compartments with individual locules > 2 cm. (**C**) Microcystic: multiple small compartments with locules ≤ 2 cm (“honeycomb” pattern). (**D**) Predominantly solid: solid component > 50% of total lesion volume

All PCLs were characterized according to the following predefined set of sonographic features [2, 3, 5, 16–19]:

- Locations of lesions: head, body, and tail of the pancreas;
- Echogenicity of lesions: anechoic, mixed echoic or hypoechoic;
- Size: maximum diameter of the lesion;
- Configuration of cyst (as Fig. 2 demonstrates):
  i. Unilocular cysts: a single cystic compartment without internal septations (with/without solid components);
  ii. Macrocystic: multiple cystic compartments with individual locules > 2 cm [2, 19] (with/without solid components);
  iii. Microcystic: multiple cystic compartments with individual locules ≤ 2 cm [2, 19], often described as a “honeycomb” pattern;
  iv. Predominantly solid: predominantly solid with minor cystic components (solid component > 50% of the total lesion volume);
- Presence of solid contents and size of solid contents;
- Presence of septation/wall thickening (maximum thickness ≥ 3 mm);
- Presence of calcification;
- Connection to the main pancreatic duct (MPD);
- MPD dilation (> 5 mm) and width of MPD;
- Common bile duct (CBD) dilation (> 7 mm) and width of the CBD;
- Vascularity on colour Doppler.

### Statistical analysis

Continuous variables are presented as the means ± SDs or medians (interquartile ranges) according to the distribution of the data, and categorical variables are presented as percentages (%). In the univariate analysis, the chi-square test or Fisher’s exact test was used to analyse the categorical variables, whereas Student’s t test or the rank-sum test was used to examine the continuous variables. Least absolute shrinkage and selection operator (LASSO) logistic regression analysis was used to identify the potential predictive features of malignancy in PCL patients. Moreover, the optimal value of λ was determined via tenfold cross-validation. Multivariate logistic regression analysis was applied to screen the statistically significant predictors and construct a nomogram. Multicollinearity among the final model predictors was assessed using the variance inflation factor (VIF). A VIF < 5 was considered indicative of acceptable collinearity levels. The effects sizes are presented as odds ratios (ORs) with 95% confidence intervals (95% CIs) and *P* values. The performance of the nomogram was assessed via receiver operating characteristic (ROC) curves and calibration curves. For internal validation, Harrell’s concordance index (C-index) and K-fold cross-validation were performed. Classification metrics including sensitivity, specificity, positive predictive value (PPV), negative predictive value (NPV), and accuracy were also calculated. Decision curve analysis (DCA) was also performed to determine the net benefit threshold of prediction. The results with a *P* value of < 0.05 were considered significant. All the statistical analyses were performed via R software (version 4.4.1).

## Results

### Interobserver agreement

The interobserver agreement between the two radiologists was summarized in Supplementary File 1, with Cohen’s κ values ranging from 0.536 (configuration of cyst) to 1.000 (calcification and CBD dilation) for categorical variables, and ICC values ranging from 0.696 (size of solid content) to 1.000 (width of CBD) for continuous measurements. Overall, the agreement was moderate to almost perfect, supporting the reliability of the imaging assessments.

### Patient characteristics

Table 1 shows the demographic, clinical and sonographic features of the 161 individuals with PCLs. A total of 59 benign lesions (SCN and other lesions, including pseudocysts, lymphangiomas and retention cysts) and 102 non-benign lesions (MCNs, IPMNs, SPNs, cNETs, and PDACs) were compared. The gender distribution showed a slight imbalance, with females accounting for 62.1% of the overall cohort; furthermore, there was a higher proportion of females in the benign group (69.5%) than in the non-benign group (57.8%). Age and BMI did not significantly differ between the groups. Pancreatitis was more prevalent in non-benign lesions (16.7%) than in benign lesions (5.1%). Bilirubin levels, both total and direct, were significantly elevated in the non-benign group compared with the benign group.

**Table 1:** Demographic, clinical and sonographic characteristics of PCL patients

Characteristics	Overall (*N* = 161)	Non-benign (*N* = 102)	Benign (*N* = 59)	*P*-value
**Pathology**				
MCN	39 (24.2%)	39 (38.2%)	-	
IPMN	49 (30.4%)	49 (48.0%)	-	
SPN	6 (3.7%)	6 (5.9%)	-	
cNET	5 (3.1%)	5 (4.9%)	-	
PDAC	3 (1.9%)	3 (2.9%)	-	
SCN	49 (30.4%)	-	49 (83.1%)	
Others	10 (6.2%)	-	10 (16.9%)	
**Gender (Female)**	100 (62.1%)	59 (57.8%)	41 (69.5%)	0.142
**Age (y.o.)**	53 (41, 63)	54 (40, 64)	52 (43, 59)	0.651
**BMI (kg/m²)**	23.3 ± 3.6	23.0 ± 3.6	23.9 ± 3.5	0.122
**Symptom (Yes)**	93 (57.8%)	62 (60.8%)	31 (52.5%)	0.308
**Pancreatitis (Yes)**	20 (12.4%)	17 (16.7%)	3 (5.1%)	**0.032**
**Diabetes mellitus (Yes)**	23 (14.3%)	16 (15.7%)	7 (11.9%)	0.504
**Smoking (Yes)**	38 (23.6%)	23 (22.5%)	15 (25.4%)	0.679
**Drinking (Yes)**	30 (18.6%)	21 (20.6%)	9 (15.3%)	0.402
**CEA (ng/ml)**	1.69 (1.05, 2.40)	1.70 (1.09, 2.50)	1.49 (1.03, 2.10)	0.570
**CA199 (U/ml)**	12 (7, 22)	13 (8, 22)	10 (6, 20)	0.105
**TBil (µmol/L)**	11.3 (9.5, 14.4)	11.9 (9.6, 15.5)	10.5 (9.1, 13.3)	**0.048**
**DBil (µmol/L)**	3.40 (2.80, 4.70)	3.70 (2.90, 5.15)	3.20 (2.55, 3.80)	**0.007**
**Location (tail/body/head)**	61/46/54	34/29/39	27/17/15	0.187
**Echogenicity (ane/mixed/hypo)**	110/36/15	73/21/8	37/15/7	0.480
**Size (cm)**	4.70 (3.20, 6.20)	4.70 (3.20, 6.28)	4.40 (3.25, 6.10)	0.846
**Configuration of cyst**				**< 0.001**
Unilocular cyst	46 (28.6%)	36 (35.3%)	10 (16.9%)	
Microcystic	34 (21.1%)	11 (10.8%)	23 (39.0%)	
Macrocystic	47 (29.2%)	31 (30.4%)	16 (27.1%)	
Predominantly solid	34 (21.1%)	24 (23.5%)	10 (16.9%)	
**Solid content (Yes)**	54 (33.5%)	43 (42.2%)	11 (18.6%)	**0.002**
**Size of solid content (cm)**	0.00 (0.00, 2.30)	0.00 (0.00, 2.60)	0.00 (0.00, 0.00)	**0.005**
**Septation/wall thickening (Yes)**	24 (14.9%)	18 (17.6%)	6 (10.2%)	0.199
**Connection to MPD (Yes)**	26 (16.1%)	22 (21.6%)	4 (6.8%)	**0.014**
**Width of MPD (mm)**	1.0 (1.0, 3.5)	1.0 (1.0, 4.0)	1.0 (1.0, 1.0)	**0.012**
**MPD dilation (>5 mm)**	27 (16.8%)	24 (23.5%)	3 (5.1%)	**0.003**
**Width of CBD (mm)**	4.0 (4.0, 4.0)	4.0 (4.0, 4.0)	4.0 (4.0, 4.0)	**0.049**
**CBD Dilation (>7 mm)**	11 (6.8%)	8 (7.8%)	3 (5.1%)	0.747
**Calcification (Yes)**	6 (3.7%)	3 (2.9%)	3 (5.1%)	0.670
**Vascularity (Yes)**	33 (20.5%)	20 (19.6%)	13 (22.0%)	0.713

n (%); Median (IQR); Mean ± SD

Pearson’s Chi-squared test; Wilcoxon rank sum test; Welch Two Sample t-test; Fisher’s exact testNote: MCN, mucinous cystic neoplasm; IPMN, intraductal papillary mucinous neoplasm; SPN, solid pseudopapillary neoplasm; cNET, cystic neuroendocrine neoplasm; PDAC, pancreatic ductal adenocarcinoma; SCN, serous cystic neoplasm; Others including pseudocyst, lymphangioma and retention cyst; TBil, total bilirubin; DBil, direct bilirubin; MPD, main pancreatic duct; CBD, common bile duct.

When examining the sonographic characteristics of lesions, the configuration of cysts displayed a significant association with malignancy; notably, microcystic structures were more common in benign lesions (39.0%). The presence of solid content was significantly more common in premalignant or malignant cases (42.2%), and the size of the solid content was larger. Additionally, non-benign lesions had more presences of connection to MPD (21.6%), MPD dilation (23.5%) and wider CBD.

**Fig. 3:**
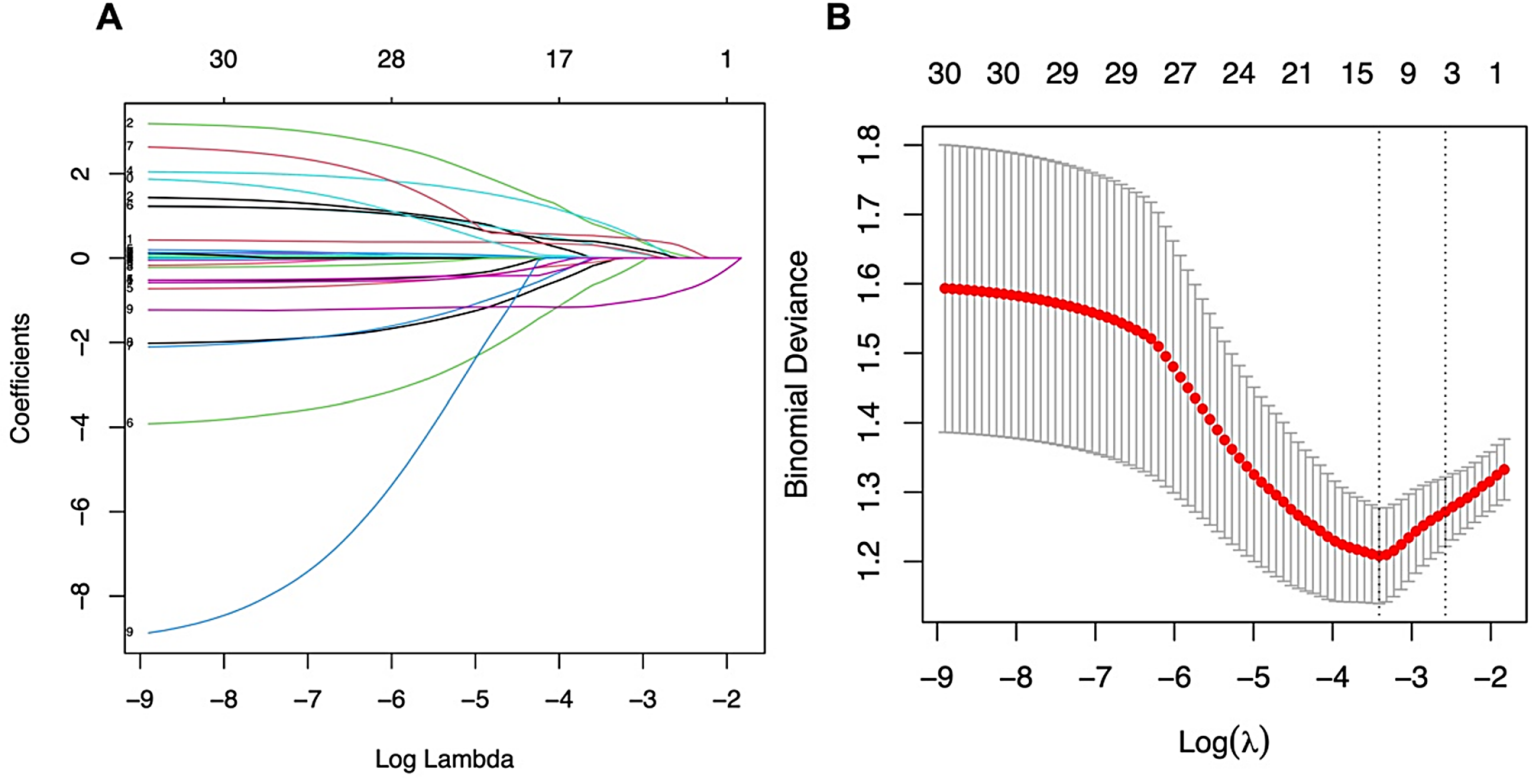
Feature selection via the LASSO binary logistic regression model. (**A**) Log (Lambda) values of the 26 features in the LASSO model. A coefficient profile plot was produced against the log (lambda) sequence. (**B**) Parameter selection in the LASSO model used tenfold cross-validation via the minimum criterion. Partial likelihood deviation (binomial deviation) curves and logarithmic (lambda) curves were plotted. The minimum standard and 1se (1-SE standard) of the minimum standard were used to draw a vertical dashed line at the optimal value. The optimal lambda produced 11 nonzero coefficients. Abbreviations: LASSO, least absolute shrinkage and selection operator; SE, standard error

Among the 26 features collected from patients, 11 features were chosen on the basis of nonzero coefficients calculated via LASSO logistic regression analysis (Fig. 3). These selected features included pancreatitis, smoking, TBil, location of the lesion, echogenicity, configuration of cyst, solid content, septation/wall thickening, connection to MPD, MPD dilation and width of the CBD. These features were subsequently included in multivariate logistic regression analysis.

### Predictive model

Multivariate logistic regression analysis revealed that echogenicity, configuration of cysts, solid content and septation/wall thickening were independent predictive factors of the malignancy of PCLs (Table 2). Hypoechoic features indicated a substantial decrease in malignancy risk. Configuration of the cysts revealed that individuals with unilocular cysts faced significantly higher risks. Moreover, the presence of solid content and septation/wall thickening substantially increased the risk of malignancy. Multicollinearity assessment showed all VIF values were below 5 (Echogenicity: 1.53, Configuration of cyst: 1.39, Solid content: 2.16, Septation/wall thickening: 1.12), confirming no significant multicollinearity among the final predictors.

**Fig. 4:**
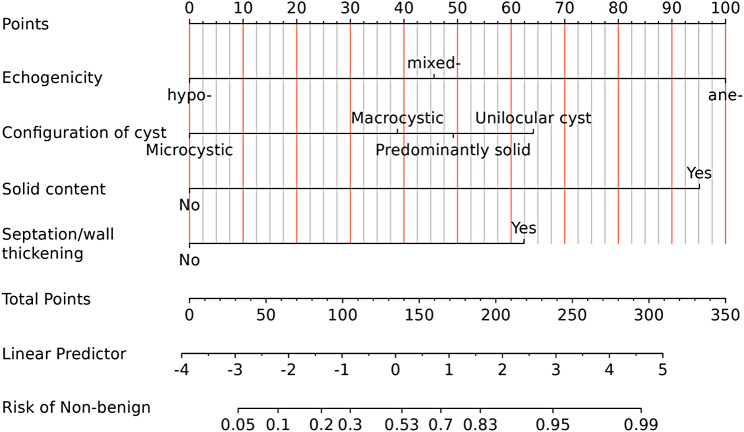
Nomogram for predicting the malignant potential of PCLs. To use the nomogram, locate the patient’s value for each predictor, draw a line upward to the ‘Points’ axis to determine the score for each variable. Sum the scores for all variables and locate the total score on the ‘Total Points’ axis. Draw a line straight down to find the predicted probability of the lesion being non-benign. To illustrate the practical application of the nomogram, three worked examples from our study cohort — representing low-risk, intermediate-risk, and high-risk cases — are provided in Supplementary File 2, with step-by-step score calculations and risk classification

These independent predictors were used to develop a nomogram, as shown in Fig. 4.

**Table 2:** Prediction factors for risk of malignancy in PCLs patients

Characteristic	β^1^	OR (95% CI)^2^	*P*-value^3^
**Echogenicity**			
ane-	—	—	
mixed-	-1.558	0.21 (0.04, 1.11)	0.066
hypo-	-2.864	0.06 (0.01, 0.57)	0.015*
**Configuration of cyst**			
Microcystic	—	—	
Unilocular cyst	1.838	6.28 (1.84, 21.50)	0.003**
Macrocystic	1.111	3.04 (0.96, 9.57)	0.058
Predominantly solid	1.410	4.09 (0.49, 34.33)	0.194
**Solid content**	2.725	15.26 (2.49, 93.65)	0.003**
**Septation/wall thickening**	1.788	5.98 (1.74, 20.57)	0.005**

^1^ Regression coefficient. ^2^ OR = Odds Ratio, CI = Confidence Interval. ^3^ *P < 0.05; **P < 0.01; ***P < 0.001

### Performance and validation of the nomogram

**Fig. 5:**
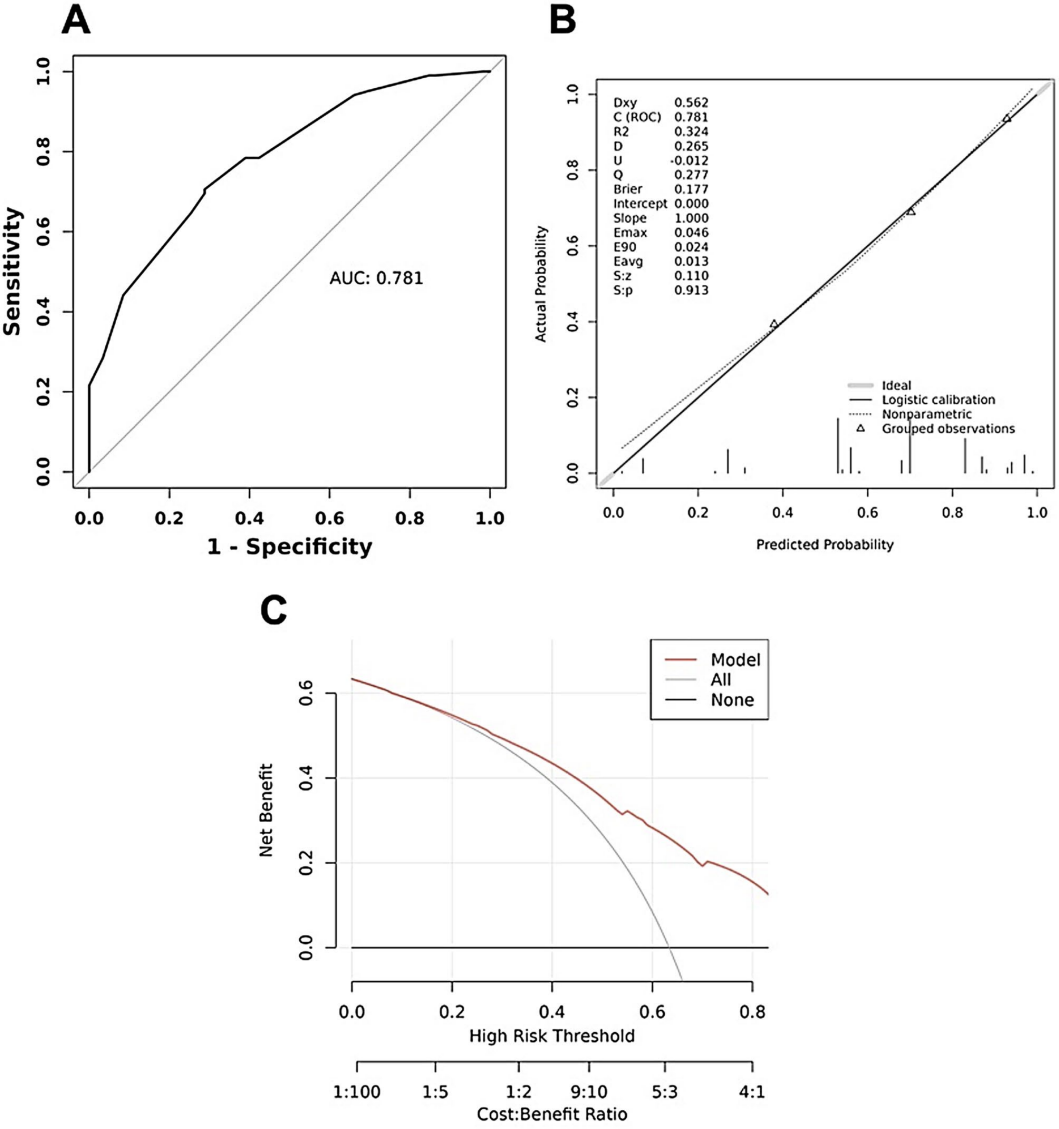
Performance of the nomogram prediction model. (**A**) Receiver operating characteristic curve of the nomogram. (**B**) Calibration curves of the nomogram. (**C**) Decision curve analysis of the nomogram

As shown in Fig. 5A, the ROC curve analysis of the abovementioned 4 variables yielded an area under the curve (AUC) value of 0.781 (95% CI: 0.711–0.851). The maximum Youden’s J index was 0.418, corresponding to an optimal threshold of 0.579 for the predicted malignancy probability. At this threshold, the model achieved the following performance metrics (Table 3): Sensitivity: 0.706 (95% CI: 0.611–0.786), Specificity: 0.712 (95% CI: 0.586–0.812), PPV: 0.809 (95% CI: 0.715–0.877), NPV: 0.583 (95% CI: 0.468–0.690), and Overall accuracy: 0.708 (95% CI: 0.634–0.773). The calibration plots are plotted in Fig. 5B, which demonstrate that the nomogram was relatively close to the ideal curve, thus indicating that the predicted results were consistent with the actual findings. Tenfold cross-validation yielded a C-index of 0.749, indicating that the model has good discrimination ability. Additionally, the Hosmer–Lemeshow test yielded *P* = 0.945, suggesting that the model showed a good fit.

Figure 5C displays the DCA curves related to the nomogram. A high-risk threshold probability indicates the chance of significant discrepancies in the model’s prediction when clinicians encounter major flaws while utilizing the nomogram for decision-making purposes. This research shows that the nomogram offers substantial net benefits for clinical application.

**Table 3:** Performance metrics of the model

Metrics	Full-Dataset (95% CI^1^)	Ten-Fold CV^2^ (Mean ± SD^3^, 95% CI^1^)
**AUC**	0.781 (0.711–0.851)	0.749 ± 0.112 (NA)
**Sensitivity**	0.706 (0.611–0.786)	0.676 ± 0.182 (0.581–0.759)
**Specificity**	0.712 (0.586–0.812)	0.661 ± 0.081 (0.534–0.769)
**PPV**	0.809 (0.715–0.877)	0.775 ± 0.067 (0.678–0.85)
**NPV**	0.583 (0.468–0.690)	0.542 ± 0.178 (0.427–0.652)
**Accuracy**	0.708 (0.634–0.773)	0.671 ± 0.117 (0.595–0.739)
**Optimal Threshold**	0.579	0.605 ± 0.031

^1^ CI = Confidence Interval. ^2^CV = Cross-validation. ^3^ SD = standard deviations

To explore the model’s clinical utility across different risk thresholds, we evaluated performance parameters at five clinically relevant cutoff values (Table 4). The high-sensitivity threshold of 0.530 maintained 94.1% sensitivity with an NPV of 0.769 (95% CI: 0.579–0.890), indicating that only 6 of 102 non-benign lesions (5.9%) would be missed; this threshold is suitable for initial screening where minimizing missed diagnoses is paramount. The Youden-optimal threshold of 0.579 provided the best balance between sensitivity (70.6%) and specificity (71.2%), with a PPV of 0.809 (95% CI: 0.715–0.877) and NPV of 0.583 (95% CI: 0.468–0.690). At the high-specificity threshold of 0.830, the model achieved 91.5% specificity with a PPV of 0.900 (95% CI: 0.786–0.957), which may be suitable for pre-surgical decision-making where confirmation of non-benign status is critical.

**Table 4:** Model performance at different risk thresholds

Threshold	Sensitivity	Specificity	PPV (95% CI)	NPV (95% CI)	Accuracy	Youden’s J	Clinical Scenario
0.319	95.1%	30.5%	0.703 (0.622–0.773)	0.783 (0.581–0.903)	71.4%	0.256	Screening (≥ 95% sensitivity)
0.530	94.1%	33.9%	0.711 (0.630–0.781)	0.769 (0.579–0.890)	72.0%	0.280	Screening (≥ 90% sensitivity)
0.579*	**70.6%**	**71.2%**	**0.809** **(0.715–0.877)**	**0.583** **(0.468–0.690)**	**70.8%**	**0.418**	**Balanced** **(Youden optimal)**
0.700	64.7%	74.6%	0.815 (0.717–0.884)	0.550 (0.441–0.654)	68.3%	0.393	Moderate-risk triage
0.830	44.1%	91.5%	0.900 (0.786–0.957)	0.486 (0.396–0.578)	61.5%	0.356	Pre-surgical (≥ 90% specificity)

* Youden optimal threshold. PPV = positive predictive value; NPV = negative predictive value; 95% CI calculated using Wilson method

### Sensitivity analysis: comparison with combined models

To evaluate the incremental value of clinical features, we compared the 4-feature ultrasound-only model with a combined model incorporating all 11 LASSO-selected variables (including pancreatitis history, smoking, TBil, and additional ultrasound measurements). The combined model yielded a higher apparent AUC (0.829, 95% CI: 0.765–0.894) compared with the ultrasound-only model (0.781, 95% CI: 0.711–0.851; DeLong *P* = 0.042). However, 10-fold cross-validated AUCs were comparable (0.750 vs. 0.749), indicating that the apparent improvement did not generalize and was likely attributable to overfitting with the larger feature set. The ultrasound-only model was therefore retained as the final model for its parsimony and point-of-care applicability.

## Discussion

Our study developed a nomogram based on TAUS features to differentiate between non-benign and benign PCLs in a cohort of 161 patients. The independent predictors identified in our study were echogenicity, configuration of cysts, solid content, and septation/wall thickening. The model demonstrated moderate discriminative performance, with an AUC of 0.781. Internal validation via tenfold cross-validation (C-index = 0.749) confirmed the model’s generalizability, suggesting its potential utility in clinical practice.

Compared with published prediction models for PCLs across different imaging modalities, our TAUS-based nomogram (AUC = 0.781) demonstrated competitive performance. CT-based radiomics models have reported AUCs ranging from 0.817 to > 0.93 for PCL subtype differentiation and malignancy prediction [20, 21], and CT radiomics-based machine learning for IPMN grading achieved an AUC of 0.85 [22]. MRI-based IPMN nomograms have shown comparable discriminative ability (C-index = 0.803) [23], while EUS-based models incorporating clinical and morphological features achieved higher AUCs of 0.938–0.951 [24], likely attributable to the superior spatial resolution of EUS. For conventional ultrasound, a recent deep learning model differentiating SCN from MCN achieved AUCs of 0.866–0.904 [25], supporting the diagnostic potential of TAUS-based approaches. TAUS offers distinct advantages as a first-line screening tool: it is radiation-free, widely available, low-cost, and easily repeatable, making it particularly suited for initial detection and longitudinal surveillance of PCLs [26, 27]. Importantly, our nomogram relies solely on conventional B-mode and Doppler features without requiring advanced techniques such as radiomics or deep learning, enhancing its clinical accessibility. Our model is intended to complement, not replace, CT/MRI/EUS in the diagnostic pathway — specifically by identifying patients who may benefit from further cross-sectional imaging or EUS evaluation.

The four independent predictors identified in our study are broadly consistent with prior literature on PCL characterization. Solid content and septation/wall thickening have been recognized as worrisome features in the 2019 WGO Global Guidelines [16] and the revised Fukuoka guidelines [3]. The roles of cyst configuration and echogenicity in our model warrant further discussion, as described below.

For the predictor of configuration of cyst, the unilocular cysts, macrocystic and predominantly solid lesions were more likely to be non-benign PCLs compared to microcystic lesions. The classification method for this feature was partially different from previous studies. Fan et al. classified PCLs into four morphologic types according to contrast-enhanced ultrasound (CEUS) methods: type I, unilocular cysts; type II, microcystic lesions; type III, macrocystic lesions; and type IV, cystic lesions with solid components or irregular thickening of the cystic wall or septa [19]. Wang et al. analysed the pathological results of 45 PCLs via this method and reported that types I, II, III and IV were most likely simple cysts, SCNs, MCNs and solid tumours (pancreatic carcinomas and SPNs), respectively [28]. However, their method could not encompass the full range of sonographic appearances observed in our cohort. Additionally, their method was based on CEUS images, which are different from TAUS images. Therefore, we modified this method and combined the features of the configuration of cyst with other features to better describe the lesions. For example, solid content and septation/wall thickening were treated as separate independent variables rather than components of the configuration classification.

Moreover, regarding echogenicity, non-benign lesions tend to be anechoic or mixed echoic. Hypoechogenicity was identified as a protective factor against non-benign pathology in our model (OR = 0.06, *P* = 0.015), with the anechoic pattern serving as the reference category. This finding warrants careful interpretation. Among the 15 hypoechoic lesions in our cohort, 7 (46.7%) were histologically confirmed as SCN—all classified as benign—while the remaining 8 comprised IPMN (*n* = 4), SPN (*n* = 2), and cNET (*n* = 2) (Supplementary Table S3). The benign hypoechoic lesions (SCN) tended to be smaller (3.3 ± 1.3 cm vs. 4.2 ± 1.8 cm), had lower CA199 levels (4.9 ± 2.9 vs. 39.4 ± 88.0 U/mL), and a lower rate of MPD dilation (14.3% vs. 37.5%) compared with the non-benign hypoechoic lesions. SCN often consists of densely packed microcysts that may be too small to resolve individually on TAUS, creating a homogeneous hypoechoic appearance rather than the typical microcystic pattern. Similarly, SPN and cNET are predominantly solid tumors that can also manifest as hypoechoic on TAUS. In contrast, anechoic lesions—the predominant pattern in our cohort (110/161, 68.3%)—had a high non-benign rate (73/110, 66.4%), as this pattern is characteristic of mucin-filled cystic compartments in MCN and IPMN. Thus, the low OR reflects a relative rather than absolute protective effect. Despite these findings, clinicians should note that TAUS assessment of internal echogenicity can be limited by bowel gas, obesity, and lesion location [12], further underscoring the importance of combining multiple sonographic features for prediction.

The nomogram developed herein has several clinical implications. It provides a quantitative, point-of-care tool for predicting the malignant potential of PCLs at initial TAUS evaluation, without requiring advanced imaging equipment or specialized software. Given that PCL patients may require long-term imaging surveillance, the non-invasive, radiation-free, and repeatable nature of TAUS makes it particularly advantageous for serial monitoring [18, 29]. Although TAUS is not currently the standard examination for the diagnosis of PCLs, the performance of this nomogram suggests that the combination of these specific TAUS features can be a valuable tool for risk stratification.

Based on the dual-threshold analysis, we propose a TAUS-based triage framework. Patients with a nomogram-predicted probability below 0.530 (high-sensitivity threshold: sensitivity 94.1%, NPV 0.769) may be considered for conservative surveillance with periodic TAUS every 6–12 months, as the probability of missed non-benign pathology is only 5.9%. Patients with predicted probabilities between 0.530 and 0.830 fall into an intermediate-risk zone and should be referred for further evaluation with CT, MRI, or EUS to refine the diagnosis. Patients exceeding the high-specificity threshold of 0.830 (specificity 91.5%, PPV 0.900) should be prioritized for multidisciplinary evaluation and surgical consultation. This stratified approach aims to reduce unnecessary invasive procedures for low-risk patients while maintaining high sensitivity for detecting non-benign lesions. The correspondence between nomogram Total Points and the three-tier risk categories is detailed in Supplementary File 2, Table S2-2. Using the high-sensitivity threshold (predicted probability = 0.530, Total Points ≈ 139) and the high-specificity threshold (predicted probability = 0.830, Total Points ≈ 190), clinicians can directly classify patients based on nomogram scores: Total Points < 139 (low risk, routine surveillance), 139–190 (intermediate risk, further workup recommended), and > 190 (high risk, surgical consultation).

Our study has several limitations that should be acknowledged. First, this was a single-center retrospective study without external validation. Due to the limited sample size, we employed 10-fold cross-validation rather than a train-test split. PCLs requiring surgical resection are relatively uncommon, making it challenging to assemble a sufficient cohort. We plan to conduct prospective validation at our institution, followed by multi-center external validation; until then, the nomogram should be considered exploratory. Second, only surgically resected, pathologically confirmed cases were included, introducing spectrum bias that may have enriched the cohort with higher-risk lesions and overestimated the model’s positive predictive value. Future studies should include patients across the full clinical spectrum, including those under surveillance. Third, the model was derived from a single pre-operative TAUS examination without incorporating longitudinal morphological changes such as diameter growth, new onset of solid components or cyst wall thickening. Future studies incorporating serial TAUS assessments could leverage this modality’s non-invasive, radiation-free, and repeatable nature for dynamic risk monitoring [27].

## Conclusions

This study explored the value of TAUS in predicting the malignant potential of PCLs. The incorporation of TAUS features into a nomogram may offer a potential non-invasive tool to assist clinicians in risk stratification during the initial evaluation and follow-up of PCL patients. Further external validation is needed before clinical implementation.

## Supplementary Information

Below is the link to the electronic supplementary material.

Supplementary Material 1

Supplementary Material 2

Supplementary Material 3

## Data Availability

The datasets used and analyzed during the current study are available from the corresponding author on reasonable request.

## Abbreviations

AUC: Area under the curve
BMI: Body mass index
CBD: Common bile duct
CEA: Carcinoembryonic antigen
CEUS: Contrast-enhanced ultrasound
CI: Confidence interval
cNET: Cystic neuroendocrine neoplasm
CT: Computed tomography
DCA: Decision curve analysis
IPMN: Intraductal papillary mucinous neoplasm
LASSO: Least absolute shrinkage and selection operator
MCN: Mucinous cystic neoplasm
MPD: Main pancreatic duct
MRI: Magnetic resonance imaging
OR: Odds ratio
PCL: Pancreatic cystic lesions
PDAC: Pancreatic ductal adenocarcinoma
ROC: Receiver operating characteristic
SCN: Serous cystic neoplasm
SPN: Solid pseudopapillary neoplasm
TAUS: Trans-abdominal ultrasound
TBil/DBil: Total/ Direct bilirubin
