# Supplementary material for: A nomogram for malignancy prediction of pancreatic cystic lesions based on trans-abdominal ultrasound features
Source: BMC Med Imaging. 2026 Apr 2;26:249. doi: 10.1186/s12880-026-02325-z (PMC13169871; doi:10.1186/s12880-026-02325-z)
Supplement: Supplementary file 1 — Supplementary Material 1 [file 12880_2026_2325_MOESM1_ESM.docx]

**Supplementary File 1**

**Interobserver Agreement for TAUS Feature Assessment**

Two radiologists independently reviewed all TAUS images blinded to pathological results and each other's assessments. Cohen's kappa (κ) was calculated for categorical variables and intraclass correlation coefficient (ICC, two-way random, single measures, absolute agreement) for continuous variables. Agreement was interpreted using the Landis and Koch scale for κ (<0.00 = poor, 0.00–0.20 = slight, 0.21–0.40 = fair, 0.41–0.60 = moderate, 0.61–0.80 = substantial, 0.81–1.00 = almost perfect) and the Koo and Li classification for ICC (<0.50 = poor, 0.50–0.75 = moderate, 0.75–0.90 = good, ≥0.90 = excellent). N = 160 patients with complete paired assessments.

**Table S1. Interobserver Agreement for Categorical TAUS Features (Cohen's Kappa)**

| **Variable** | **Categories** | **Agreement n/N (%)** | **Cohen's κ** | **Interpretation** |
| --- | --- | --- | --- | --- |
| Location of lesion | 4 | 149/160 (93.1%) | 0.899 | Almost perfect |
| Echogenicity | 3 | 151/160 (94.4%) | 0.880 | Almost perfect |
| Configuration of cyst | 4 | 106/160 (66.2%) | 0.536 | Moderate |
| Solid content | 2 | 142/160 (88.8%) | 0.744 | Substantial |
| Septation/wall thickening | 2 | 147/160 (91.9%) | 0.638 | Substantial |
| Calcification | 2 | 160/160 (100.0%) | 1.000 | Almost perfect |
| Connection to MPD | 2 | 151/160 (94.4%) | 0.760 | Substantial |
| MPD dilation | 2 | 144/160 (90.0%) | 0.712 | Substantial |
| CBD dilation | 2 | 160/160 (100.0%) | 1.000 | Almost perfect |
| Vascularity | 2 | 158/160 (98.8%) | 0.962 | Almost perfect |

**Table S2. Interobserver Agreement for Continuous TAUS Measurements (ICC)**

| **Variable** | **Reader 1 (mean ± SD)** | **Reader 2 (mean ± SD)** | **ICC (2,1)** | **95% CI** | **Interpretation** |
| --- | --- | --- | --- | --- | --- |
| Cyst size (cm) | 5.00 ± 2.50 | 4.91 ± 2.57 | 0.978 | 0.97–0.98 | Excellent |
| Size of solid content (cm) | 1.26 ± 2.20 | 1.01 ± 1.81 | 0.696 | 0.61–0.77 | Moderate |
| Width of MPD (mm) | 2.98 ± 4.14 | 2.98 ± 4.15 | 0.999 | ~1.00 | Excellent |
| Width of CBD (mm) | 4.56 ± 1.75 | 4.56 ± 1.75 | 1.000 | —* | Perfect |

** Width of CBD showed perfect agreement between raters (identical values for all patients); confidence interval could not be computed.
ICC = intraclass correlation coefficient; MPD = main pancreatic duct; CBD = common bile duct; TAUS = transabdominal ultrasound.*
