# Supplementary material for: A nomogram for malignancy prediction of pancreatic cystic lesions based on trans-abdominal ultrasound features
Source: BMC Med Imaging. 2026 Apr 2;26:249. doi: 10.1186/s12880-026-02325-z (PMC13169871; doi:10.1186/s12880-026-02325-z)
Supplement: Supplementary file 2 — Supplementary Material 2 [file 12880_2026_2325_MOESM2_ESM.docx]

# Supplementary File 2

## Worked Examples of Nomogram Application for Score Calculation and Risk Stratification


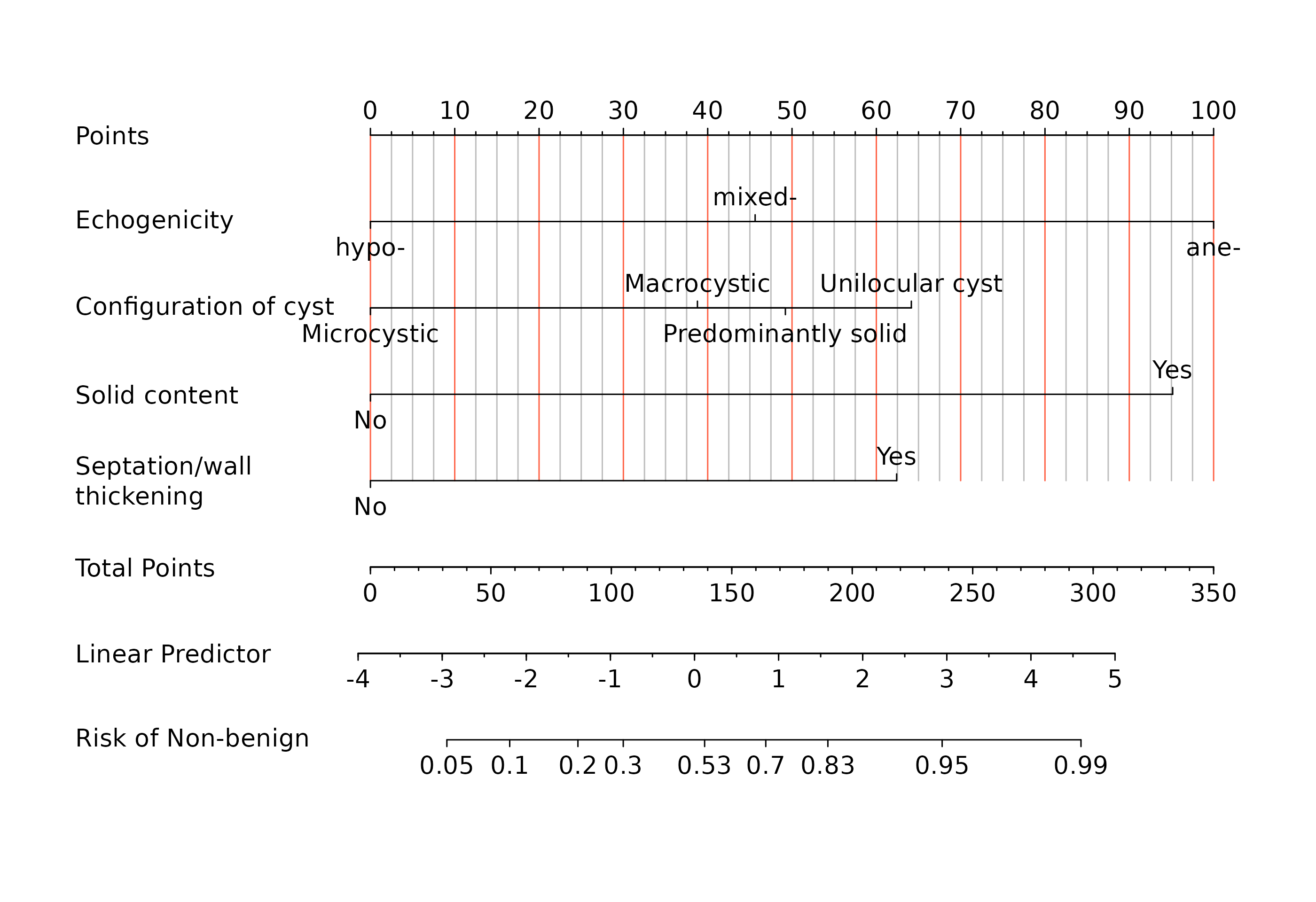


To facilitate clinical application of the nomogram (Figure 4), we present three worked examples from our study cohort demonstrating the step-by-step process of score calculation and risk prediction. These examples cover the full spectrum of predicted risk: low, intermediate, and high. For each case, the user should:

1. Step 1: For each predictor variable, locate the patient's value on the corresponding axis of the nomogram.
2. Step 2: Draw a vertical line upward to the "Points" axis at the top and read the score for that variable.
3. Step 3: Sum the scores for all four variables to obtain the Total Points.
4. Step 4: Locate the Total Points on the "Total Points" axis, draw a vertical line straight down through the "Linear Predictor" axis to the "Risk of Non-benign" axis, and read the predicted probability directly from the scale.
5. Step 5: Classify the patient into a risk category based on the dual-threshold framework (Table 4).

### Table S2-1. Nomogram Points Reference

The point assignments for each predictor level are summarized below. These values correspond to the nomogram scales in Figure 4.

| **Variable** | **Level** | **Points** |
| --- | --- | --- |
| Echogenicity | Hypoechoic | 0 |
|  | Mixed echoic | 46 |
|  | Anechoic (reference) | 100 |
| Configuration of cyst | Microcystic (reference) | 0 |
|  | Macrocystic | 39 |
|  | Predominantly solid | 49 |
|  | Unilocular cyst | 64 |
| Solid content | No | 0 |
|  | Yes | 95 |
| Septation/wall thickening | No | 0 |
|  | Yes | 62 |

### Table S2-2. Risk Stratification Based on Nomogram Total Points

The "Risk of Non-benign" axis on the nomogram represents the predicted probability of non-benign pathology for each individual patient. This probability is identical to the threshold values used in the model performance analysis (Table 4). When a specific probability value is chosen as a decision threshold for the entire cohort, the corresponding sensitivity, specificity, PPV, and NPV can be evaluated. Based on the dual-threshold analysis, the following three-tier risk stratification is proposed:

| **Risk Category** | **Total Points** | **Risk of Non-benign** | **Threshold Basis** | **Recommended Action** |
| --- | --- | --- | --- | --- |
| Low | < 139 | < 0.530 | High-sensitivity (Sens 94.1%, Spec 33.9%) | Routine surveillance |
| Intermediate | 139 – 190 | 0.530 – 0.830 | Between dual thresholds | Further workup (EUS / CT / MRI) |
| High | > 190 | > 0.830 | High-specificity (Sens 44.1%, Spec 91.5%) | Surgical consultation |

### Case 1: Low Risk

**Patient:** female, age 50-60, asymptomatic, located in the body of the pancreas, size 2.2 cm

**Pathology:** Benign (SCN)

**Steps 1–2: Identify predictor values and read corresponding points from the nomogram**

| **Predictor** | **Patient Value** | **Points** |
| --- | --- | --- |
| Echogenicity | Hypoechoic | 0 |
| Configuration of cyst | Microcystic | 0 |
| Solid content | No | 0 |
| Septation/wall thickening | No | 0 |
|  | **Total Points** | **0** |

**Step 3: Sum all points → Total Points =** 0

**Step 4: Read predicted probability from the nomogram → Risk of Non-benign =** 0.021 (2.1%)

*(Locate 0 on the "Total Points" axis, draw a vertical line downward to the "Risk of Non-benign" axis to read the predicted probability directly.)*

**Step 5: Risk stratification → Low risk**

Total Points = 0 < 139, corresponding to Risk of Non-benign = 0.021, which is below the high-sensitivity threshold of 0.530.

**Clinical recommendation:** This patient is classified as low risk. The predicted probability of 2.1% is well below the high-sensitivity threshold (0.530), at which the model achieves 94.1% sensitivity and an NPV of 0.769. Routine surveillance is recommended.

### Case 2: Intermediate Risk

**Patient:** female, age 50-60, symptomatic, located in the head of the pancreas, size 6.8 cm

**Pathology:** Non-benign (IPMN)

**Steps 1–2: Identify predictor values and read corresponding points from the nomogram**

| **Predictor** | **Patient Value** | **Points** |
| --- | --- | --- |
| Echogenicity | Mixed echoic | 46 |
| Configuration of cyst | Macrocystic | 39 |
| Solid content | No | 0 |
| Septation/wall thickening | Yes | 62 |
|  | **Total Points** | **147** |

**Step 3: Sum all points → Total Points =** 147

**Step 4: Read predicted probability from the nomogram → Risk of Non-benign =** 0.587 (58.7%)

*(Locate 147 on the "Total Points" axis, draw a vertical line downward to the "Risk of Non-benign" axis to read the predicted probability directly.)*

**Step 5: Risk stratification → Intermediate risk**

Total Points = 147 falls between 139 (high-sensitivity threshold, P = 0.530) and 190 (high-specificity threshold, P = 0.830).

**Clinical recommendation:** This patient is classified as intermediate risk. The predicted probability of 58.7% exceeds the high-sensitivity threshold but does not reach the high-specificity threshold. Further diagnostic workup such as EUS or cross-sectional imaging (CT/MRI) is recommended to refine the diagnosis before surgical decision-making.

### Case 3: High Risk

**Patient:** female, age 30-40, symptomatic, located in the tail of the pancreas, size 7.4 cm

**Pathology:** Non-benign (MCN)

**Steps 1–2: Identify predictor values and read corresponding points from the nomogram**

| **Predictor** | **Patient Value** | **Points** |
| --- | --- | --- |
| Echogenicity | Anechoic | 100 |
| Configuration of cyst | Macrocystic | 39 |
| Solid content | Yes | 95 |
| Septation/wall thickening | Yes | 62 |
|  | **Total Points** | **296** |

**Step 3: Sum all points → Total Points =** 296

**Step 4: Read predicted probability from the nomogram → Risk of Non-benign =** 0.990 (99.0%)

*(Locate 296 on the "Total Points" axis, draw a vertical line downward to the "Risk of Non-benign" axis to read the predicted probability directly.)*

**Step 5: Risk stratification → High risk**

Total Points = 296 > 190, corresponding to Risk of Non-benign = 0.990, which exceeds the high-specificity threshold of 0.830.

**Clinical recommendation:** This patient is classified as high risk. The predicted probability of 99.0% far exceeds the high-specificity threshold (0.830), at which the model achieves 91.5% specificity and a PPV of 0.900. Surgical consultation is strongly recommended. Notably, the markedly elevated CA19-9 (103.5 U/mL) further supports clinical concern for malignancy, although CA19-9 is not included in the nomogram model.

### Note

The three-tier risk stratification is based on the dual-threshold analysis presented in the main text (Table 4). The high-sensitivity threshold (predicted probability = 0.530) was selected to achieve ≥ 90% sensitivity, enabling safer triage of patients into low-risk versus intermediate/high-risk categories. The high-specificity threshold (predicted probability = 0.830) was selected to achieve ≥ 90% specificity, identifying patients with the highest likelihood of non-benign pathology who may benefit from early surgical consultation.

**Abbreviations:** EUS, endoscopic ultrasound; CT, computed tomography; MRI, magnetic resonance imaging; PPV, positive predictive value; NPV, negative predictive value.
