# Supplementary material for: A nomogram for malignancy prediction of pancreatic cystic lesions based on trans-abdominal ultrasound features
Source: BMC Med Imaging. 2026 Apr 2;26:249. doi: 10.1186/s12880-026-02325-z (PMC13169871; doi:10.1186/s12880-026-02325-z)
Supplement: Supplementary file 3 — Supplementary Material 3 [file 12880_2026_2325_MOESM3_ESM.docx]

**Supplementary Table S3**

**Characteristics of 15 Hypoechoic Pancreatic Cystic Lesions Stratified by Pathological Diagnosis**

|  | **SCN (n=7)** | **IPMN (n=4)** | **SPN (n=2)** | **cNET (n=2)** |
| --- | --- | --- | --- | --- |
| Group | Benign | Non-benign | Non-benign | Non-benign |
| **Demographics** |  |  |  |  |
| Gender (M/F) | 2/5 | 3/1 | 1/1 | 2/0 |
| Age (years) | 56.0 ± 8.0 | 64.0 ± 4.1 | 29.0 ± 2.8 | 56.5 ± 9.2 |
| BMI (kg/m²) | 24.2 ± 4.7 | 23.4 ± 2.8 | 25.6 ± 6.8 | 25.7 ± 2.8 |
| **Clinical Features** |  |  |  |  |
| Symptom | 3/7 (42.9%) | 3/4 (75.0%) | 0/2 (0.0%) | 1/2 (50.0%) |
| Pancreatitis | 0/7 (0.0%) | 0/4 (0.0%) | 0/2 (0.0%) | 0/2 (0.0%) |
| Diabetes | 1/7 (14.3%) | 2/4 (50.0%) | 0/2 (0.0%) | 0/2 (0.0%) |
| Smoking | 2/7 (28.6%) | 1/4 (25.0%) | 0/2 (0.0%) | 1/2 (50.0%) |
| Drinking | 1/7 (14.3%) | 2/4 (50.0%) | 0/2 (0.0%) | 0/2 (0.0%) |
| **Laboratory** |  |  |  |  |
| CEA (ng/mL) | 1.7 ± 1.3 | 2.9 ± 2.3 | 0.9 ± 0.4 | 2.1 ± 1.2 |
| CA19-9 (U/mL) | 4.9 ± 2.9 | 93.4 ± 110.7 | 4.1 ± 3.0 | 5.8 ± 5.1 |
| TBil (μmol/L) | 11.3 ± 4.5 | 14.8 ± 7.1 | 19.0 ± 6.1 | 16.4 ± 7.8 |
| DBil (μmol/L) | 3.5 ± 1.3 | 4.8 ± 2.2 | 6.4 ± 1.2 | 4.8 ± 2.8 |
| **Sonographic Features** |  |  |  |  |
| Location (head/body/tail) | 2/2/3 | 2/1/1 | 0/1/1 | 1/0/1 |
| Size (cm) | 3.3 ± 1.3 | 5.5 ± 1.0 | 2.8 ± 2.5 | 2.2 ± 0.5 |
| Configuration of cyst |  |  |  |  |
| Predominantly solid | 6/7 (85.7%) | 4/4 (100.0%) | 2/2 (100.0%) | 2/2 (100.0%) |
| Microcystic | 1/7 (14.3%) | 0 | 0 | 0 |
| Solid content | 6/7 (85.7%) | 4/4 (100.0%) | 2/2 (100.0%) | 2/2 (100.0%) |
| Size of solid content (cm)* | 3.5 ± 1.4 | 5.5 ± 1.0 | 2.8 ± 2.5 | 2.2 ± 0.5 |
| Septation/wall thickening | 0/7 (0.0%) | 0/4 (0.0%) | 0/2 (0.0%) | 0/2 (0.0%) |
| Calcification | 2/7 (28.6%) | 0/4 (0.0%) | 1/2 (50.0%) | 0/2 (0.0%) |
| Connection to MPD | 1/7 (14.3%) | 1/4 (25.0%) | 0/2 (0.0%) | 0/2 (0.0%) |
| MPD dilation | 1/7 (14.3%) | 3/4 (75.0%) | 0/2 (0.0%) | 0/2 (0.0%) |
| Width of MPD (mm) | 2.6 ± 2.9 | 7.0 ± 5.0 | 1.0 ± 0.0 | 1.0 ± 0.0 |
| CBD dilation | 1/7 (14.3%) | 1/4 (25.0%) | 0/2 (0.0%) | 0/2 (0.0%) |
| Width of CBD (mm) | 4.6 ± 1.5 | 7.8 ± 5.7 | 4.0 ± 0.0 | 4.0 ± 0.0 |
| Vascularity | 2/7 (28.6%) | 1/4 (25.0%) | 0/2 (0.0%) | 0/2 (0.0%) |

*Data are presented as mean ± SD or n/N (%).
* Size of solid content calculated only for cases with solid content present.
SCN = serous cystadenoma; IPMN = intraductal papillary mucinous neoplasm; SPN = solid pseudopapillary neoplasm; cNET = cystic neuroendocrine tumor; MPD = main pancreatic duct; CBD = common bile duct; TBil = total bilirubin; DBil = direct bilirubin.*
